# Angioedema due to acquired C1-inhibitor deficiency: spectrum and treatment with C1-inhibitor concentrate

**DOI:** 10.1186/s13023-019-1043-3

**Published:** 2019-03-13

**Authors:** Konrad Bork, Petra Staubach-Renz, Jochen Hardt

**Affiliations:** 1 Department of Dermatology, Johannes Gutenberg University, Langenbeckstr. 1, 55131 Mainz, Germany; 2 Department of Medical Psychology and Medical Sociology, Johannes Gutenberg University, Mainz, Germany

**Keywords:** Acquired angioedema, C1-inhibitor deficiency, Non-Hodgkin lymphoma, C1-inhibitor concentrate, C1-inhibitor antibodies

## Abstract

**Background:**

Acquired angioedema due to C1-inhibitor (C1-INH) deficiency (AAE-C1-INH) is a serious condition that may result in life-threatening asphyxiation due to laryngeal edema. It is associated with malignant B-cell lymphoma and other disorders. The purpose of this study was to describe the characteristics and associated disorders of patients with AAE-C1-INH and assess the efficacy of plasma-derived C1-INH concentrate (pdC1-INH) in the treatment of AAE-C1-INH. Forty-four patients with AAE-C1-INH from the Angioedema Outpatient Service of Mainz were assessed for associated disorders. In 32 of these patients, the duration of swelling attacks was measured before and after treatment with pdC1-INH (Berinert® (CSL Behring, Marburg, Germany)). The time between injection and complete resolution of symptoms and treatment effectiveness was provided by the patients.

**Results:**

The following underlying disorders were present: monoclonal gammopathy of undetermined significance (47.7%), non-Hodgkin lymphoma (27.3%), anti-C1-INH autoantibodies alone (11.4%), and other conditions (4.5%). In 9.1% patients, no associated disorder could be found. AAE-C1-INH led to the detection of lymphoma in 75% of patients with the malignancy. Treatment with pdC1-INH shortened attacks by an average (SD) 54.4 (± 32.8) hours (*P* < 0.0001). The earlier the attack was treated, the shorter the time between injection and resolution of symptoms (*P* = 0.0149). A total of 3553 (97.7%) of the 3636 attacks were effectively treated with pdC1-INH as assessed by the patient. The mean (SD) dose per-attack was 787 (± 442) U. pdC1-INH was effective in 1246 (93.8%) of 1329 attacks in 8 patients with anti-C1-INH autoantibodies and in 344 (99.4%) of 346 attacks in 6 patients without autoantibodies. The average (SD) dose per effectively treated attack was 1238.4 (± 578.2) U in patients with anti-C1-INH autoantibodies and 510.2 (± 69.1) U in patients without autoantibodies.

**Conclusions:**

pdC1-INH is highly effective in treating AAE-C1-INH patients and is also effective in the vast majority of attacks in patients with anti-C1-INH autoantibodies. It is fast-acting and reduces attack duration.

## Background

Acquired angioedema due to C1-inhibitor (C1-INH) deficiency (AAE-C1-INH) or angioedema due to acquired C1-INH deficiency is characterized by acquired deficiency of C1-INH, recurrent angioedema, and hyperactivation of the complement pathway. Patients have recurrent swellings or attacks of the skin (face, extremities, and genitals) and severe abdominal attacks, sometimes with diarrhea and vomiting, due to edema of the gastrointestinal mucosa. They may also present with potentially life-threatening edema of the upper respiratory tract, oral mucosa and tongue. Death by asphyxiation has been reported [1–3]. Attacks usually last from 2 to 5 days without any apparent trigger [4]. There is no epidemiological data for AAE-C1-INH available but prevalence has been estimated to range between 1:100,000 and 1:500,000 [4].

AAE-C1-INH may be associated with B-cell abnormalities such as malignant lymphoma, monoclonal gammopathy of undetermined significance (MGUS), and anti-C1-INH autoantibodies. Although functional C1-INH is produced in AAE-C1-INH, it is thought that these associated disorders can lead to a deficiency of the C1-INH protein [5–8]. The deficiency of C1-INH leads to inappropriate activation of the contact-kinin system, release of bradykinin, increased vascular permeability and angioedema [9, 10].

Diagnosis of AAE-C1-INH generally occurs after 40 years of age [11]. There is no genetic association or family history of angioedema in AAE-C1-INH; this is in contrast to the types of hereditary angioedema (HAE) which are caused by or associated with specific mutations in the genes coding for C1-INH, factor XII, plasminogen or angiopoietin [12–15]. Patients with AAE-C1-INH have low plasma levels of C1-INH (functional and antigenic) and C4 which are usually below 50% of normal. Unlike HAE-C1-INH patients, the majority of AAE-C1-INH patients also have reduced C1q levels. The presence of anti-C1-INH autoantibodies and the absence of C1-INH genetic mutations can aid in the diagnosis of AAE-C1-INH [4].

Currently, there is no approved treatment for AAE-C1-INH. HAE-C1-INH therapies such as plasma-derived C1-INH concentrate (pdC1-INH) and the bradykinin B2 receptor antagonist, icatibant, are used to resolve AAE-C1-INH attacks [16]. It is generally supposed that these on-demand treatments are most effective when administered early in the attack [17]. However, published information on the efficacy and safety of AAE-C1-INH treatments is limited and has not been studied systematically.

Therefore, the aim of this observational, retrospective study was to: (1) describe the characteristics and associated disorders of patients with AAE-C1-INH, (2) assess the efficacy of pdC1-INH in the treatment of AAE-C1-INH patients (3) assess the effect of time to injection, dose and anti-C1-INH autoantibodies on pdC1-INH efficacy.

## Results

### Patient characteristics

Patients with a confirmed diagnosis of AAE-C1-INH attending our outpatient clinic, Department of Dermatology, University of Mainz, Germany (AOSM), were eligible for this observational study. In total, 44 patients with AAE-C1-INH were identified and their health records were reviewed and analyzed. The prevalence of AAE-C1-INH compared with that of HAE-C1-INH in the AOSM was 1:9.3. The majority (61%) of patients were female and mean (SD) age at onset of recurrent angioedema of AAE-C1-INH was 56.2 (± 14.8) years (Table 1). Five (11.4%) of the 44 patients were aged under 40 years when the recurrent angioedema started. Two female patients had onset at 21 and 30 years and had anti-C1-INH autoantibodies; another male and female patient had onset at 37 and 39 years, respectively and had MGUS. The fifth patient had no associated disorders. In these 5 patients, molecular genetic testing for a mutation in *SERPING1* was negative.

**Table 1:** Patient characteristics

	All patients (*N* = 44)		pdC1-INH treated patients (*N* = 32)	
Age at first angioedema (years), mean (SD)	56.2	(14.8)	56.0	(14.7)
< 40 (years), n (%)	5	(11.4)	4	(12.5)
40 - < 50 (years), n (%)	9	(20.4)	6	(13.6)
50 - < 60 (years), n (%)	11	(11.0)	7	(21.9)
60 - < 70 (years), n (%)	10	(22.7)	9	(28.1)
70 - < 80 (years), n (%)	7	(15.9)	4	(12.5)
≥80 (years), n (%)	2	(4.5)	2	(6.3)
Gender, n (%)				
Male	17	(38.6)	14	(43.8)
Female	27	(61.4)	18	(56.3)
Associated disorders, n (%)				
MGUS	21	(47.7)	15	(46.9)
Non-Hodgkin lymphoma	12	(27.3)	10	(31.3)
Splenic marginal cell lymphoma	6	(13.6)	4	(12.5)
Plasmocytoma	2	(4.5)	2	(6.3)
B-cell lymphoma	1	(2.3)	1	(3.1)
Waldenström’s macroglobulinemia	1	(2.3)	1	(3.1)
Centroblastic-centrocytic follicular lymphoma	1	(2.3)	1	(3.1)
Diffuse anaplastic large cell B-cell lymphoma	1	(2.3)	1	(3.1)
Anti-C1-INH autoantibodies with no other associated disorders ^a^	5	(11.4)	5	(15.6)
Other associated disorders	2	(4.5)	0	
None	4	(9.1)	2	(6.3)
Plasma complement, n (%)				
C1-INH function				
< normal range of 70–130%	44	(100)	32	(100)
< 50%	44	(100)	32	(100)
< 5%	28	(63.6)	21	(65.6)
C1-INH protein				
< normal range of 15.4–33.8 mg/dL	44	(100)	32	(100)
< 12 mg/dL	41	(93.2)	32	(100)
< 4.8 mg/dL	27	61.4	20	(62.5)
C4				
< normal range of 16.4–31.3 mg/dL	44	(100)	32	(100)
< 12 mg/dL	43	(97.7)	32	(100)
< 4.8 mg/dL	39	(88.6)	30	(93.8)
C1q				
< normal range of 0.1–0.25 g/L	39	(88.6)	29	(90.6)
< 0.05 g/L	26	(59.1)	21	(65.6)

^a^. an additional 3 patients had autoantibodies to C1-INH and an associated disorder

C1-INH = C1 inhibitor; pdC1-INH = plasma-derived C1-inhibitor concentrate; MGUS = monoclonal gammopathy of undetermined significance; SD = standard deviation

Clinical symptoms were abdominal attacks, skin swellings of the face, extremities and genitals as well as isolated tongue swellings and laryngeal attacks. An erythema marginatum preceded the symptoms in 2 patients. Seven patients died during the course of the study; 2 deaths were from underlying diseases. There were no angioedema-related deaths in this group.

### Associated disorders

The search for associated disorders in all 44 AAE-C1-INH patients revealed the following (Table 1):

(1) MGUS was associated with 21 (47.7%) patients: IgG (*n* = 12), IgM (*n* = 6), and IgA (*n* = 3). The mean (SD) age at onset of angioedema in patients with MGUS was 58.5 (± 13.5) years.

(2) Non-Hodgkin lymphoma was present in 12 (27.3%) patients. Of these, 11 (25.0%) patients had low-grade malignancy lymphoma: splenic marginal cell lymphoma (*n* = 6), plasmocytoma (*n* = 2), B-cell lymphoma (*n* = 1), Waldenström’s macroglobulinemia (*n* = 1), and centroblastic-centrocytic follicular lymphoma (*n* = 1). One (2.3%) patient had high-grade malignancy lymphoma: diffuse anaplastic large cell B-cell lymphoma. Six of the 12 patients with non-Hodgkin lymphoma presented with monoclonal gammopathies: IgG (*n* = 3) and IgM (*n* = 3). The mean (SD) age at onset of angioedema in patients with non-Hodgkin lymphoma was 62.4 (± 12.3) years.

(3) Five (11.4%) patients had anti-C1-INH autoantibodies and, besides recurrent angioedema, they had no other underlying associated disorder. In these patients, recurrent angioedema started at ages 21, 30, 40, 50, and 54 years giving a mean (SD) age at onset of 39 (± 13.7) years. Interestingly, patients with anti-C1-INH autoantibodies and no other disorder were notably younger at the onset of angioedema than patients with MGUS (58.5 (± 13.5) years) or malignant lymphoma (62.4 (± 12.3) years). During an observation period of mean (SD) 15.8 (± 9.4) years following anti-C1-INH autoantibody detection, no MGUS, lymphoma or other underlying disorders besides autoantibodies were found. An additional 3 patients had anti-C1-INH autoantibodies and an underlying associated disorder of MGUS (*n* = 2) and diffuse anaplastic large cell B-cell lymphoma (*n* = 1).

(4) Two (4.5%) patients had other associated disorders: breast cancer (*n* = 1) and liver failure (*n* = 1).

(5) The 4 (9.1%) remaining patients had no associated disorders such as MGUS, non-Hodgkin lymphoma, anti-C1-INH autoantibodies, cancer or liver failure. Three of the 4 patients had low C1-INH activity and protein, low C4 and low C1q in plasma. One of the 4 patients, a female, had recurrent angioedema of the extremities and lips and abdominal attacks for 17 years (from age 25 to 42 years). During this time, the patient had low C1-INH function and protein and low C4 but normal C1q. For the last 4 years, the patient has had no clinical symptoms and all 6 tests for C1-INH function and protein and C4 revealed normal results. Mutations in the *SERPING1* gene could not be identified and there was no family history of angioedema. The patient’s parents had normal C1-INH function and protein.

In 18 patients, recurrent angioedema was present more than 1 year before an associated disorder was diagnosed, in 23 patients both were simultaneously diagnosed and in 3 patients, recurrent angioedema developed later than the associated disorder. In 9 (75.0%) of 12 patients, the associated malignant lymphoma was detected following diagnosis of AAE-C1-INH. In the other 3 patients, the underlying lymphoma was already known before the first visit at the AOSM.

### Plasma complement

All 44 patients had functional and antigenic C1-INH plasma levels which were below the normal range (Table 1). C4 levels were also below the normal range in all patients; C1q levels were below normal levels in 39 (88.6%) patients.

### Prior and concomitant treatment

Prior to initiation of treatment with pdC1-INH (Berinert® (CSL Behring, Marburg, Germany)), patients received prophylactic therapy with oral prophylaxis: danazol (10 (22.7%) patients), tranexamic acid (10 (22.7%) patients) and antihistamines (1 (2.3%) patients). Acute attacks were treated with icatibant (11 (25.0%) patients), cortisone (10 (22.7%) patients) and antihistamines (2 (4.5%) patients). Rituximab (5 (11.4%) patients) and clexanes (2 (4.5%) patients), were administered for the long-term treatment of swellings. Patients spent on average 2.8 (± 4.5) stays in hospital and 0.82 (± 2.7) days in intensive care for the treatment of acute attacks before pdC1-INH treatment. Three patients had one intubation each and 2 patients had 2 intubations each. Three patients underwent a cricothyrotomy procedure.

During the pdC1-INH treatment period, patients continued to occasionally use icatibant to treat acute attacks (11 (25.0%) patients). Rituximab (5 (11.4%) patients) and clexanes (2 (4.5%) patients) were also administered during the pdC1-INH treatment phase.

### Plasma-derived C1-inhibitor concentrate treatment

#### Attacks, treatment duration and dose

**Fig. 1:**
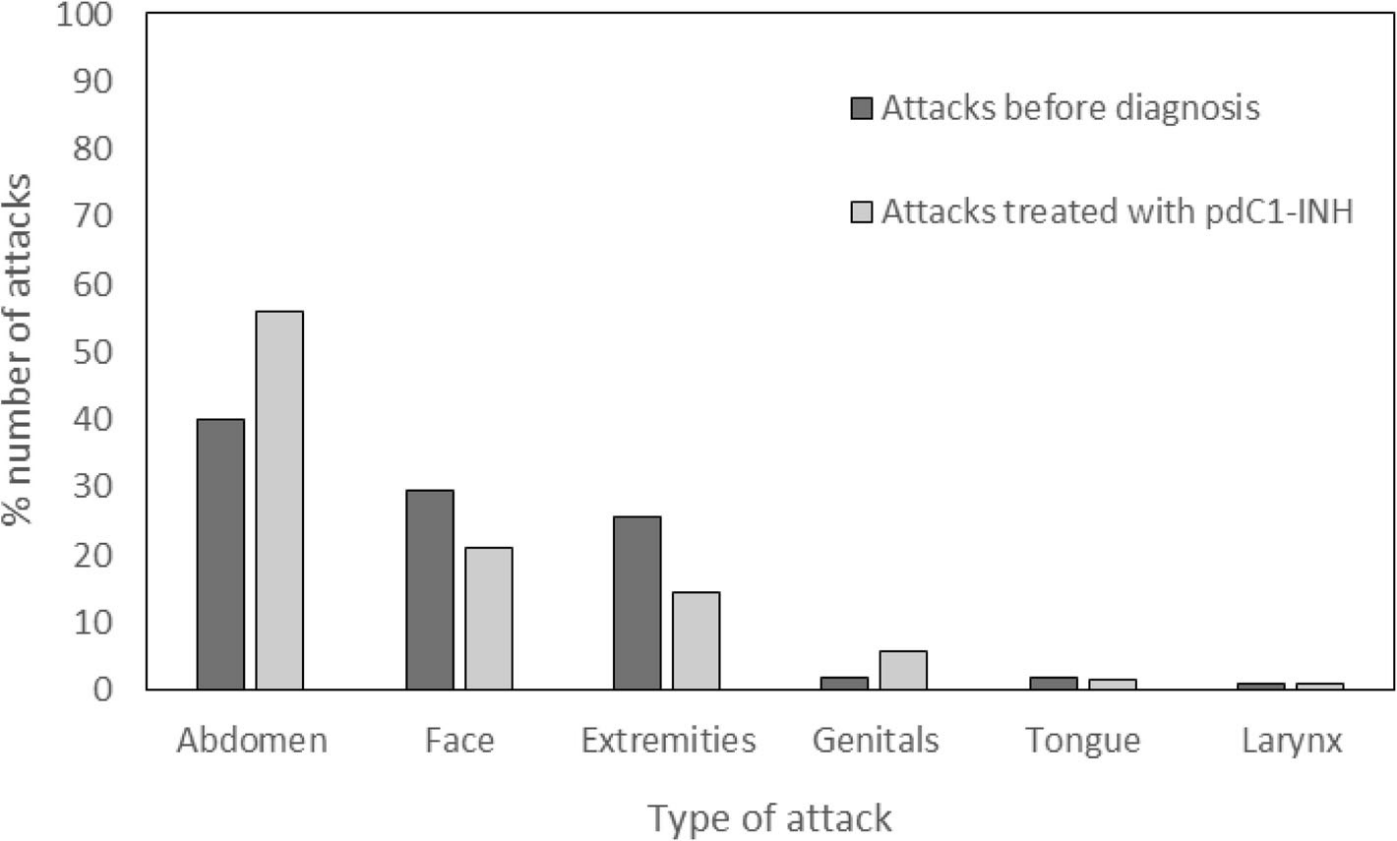
Location of attacks in acquired angioedema C1-inhibitor patients before diagnosis and after treatment with plasma-derived C1-inhibitor concentrate pdC1-INH = plasma-derived C1-inhibitor.

The pdC1-INH treatment group comprised 32 patients, the characteristics of which are listed in Table 1. A total of 1962 attacks were experienced in this group before diagnosis in the abdomen (785 (40%)), face (580 (29.6%)), extremities (504 (25.7%)), genitals (36 (1.8%)), tongue (35 (1.8%)), and larynx (22 (1.1%)). These patients received pdC1-INH for a mean (SD) 82.6 (± 80.1) months. They were treated for 3636 attacks mainly occurring in the abdomen (2522 (56.1%)), face (954 (21.2%)), and extremities (650 (14.5%)) (Table 2 and Fig. 1). A smaller number (367 (8.2%)) of attacks occurring in the genitals, tongue and larynx were also treated. pdC1-INH was administered at the 500 U dose for 2203 attacks and at the 1000 U dose for 1095 attacks (Table 2). A total of 338 attacks were treated with higher doses of 1500 U, 2000 U or 3000 U.

**Table 2:** Attacks treated with plasma-derived C1-inhibitor concentrate

	pdC1-INH concentrate treated attacks (*N* = 32)		
	Total	Mean	(SD)
Attacks treated with pdC1-INH concentrate, n	3636	113.6	(336.2)
Attacks treated by location, n			
Abdominal attacks	2522	78.8	(275.9)
Facial attacks	954	29.8	(115.5)
Extremity attacks	650	20.3	(65.8)
Genital attacks	255	8.0	(43.5)
Tongue attacks	71	2.2	(9.5)
Laryngeal attacks	41	1.3	(3.4)
Attacks treated by dose, n			
500 U	2203	68.8	(300.7)
1000 U	1095	34.2	(144.9)
1500 U	22	0.7	(2.2)
2000 U	315	9.8	(53.5)
3000 U	1	0	(0.2)

*pdC1-INH* = Plasma-derived C1-inhibitor; *SD* = Standard deviation

### Efficacy of pdC1-INH concentrate treatment

**Fig. 2:**
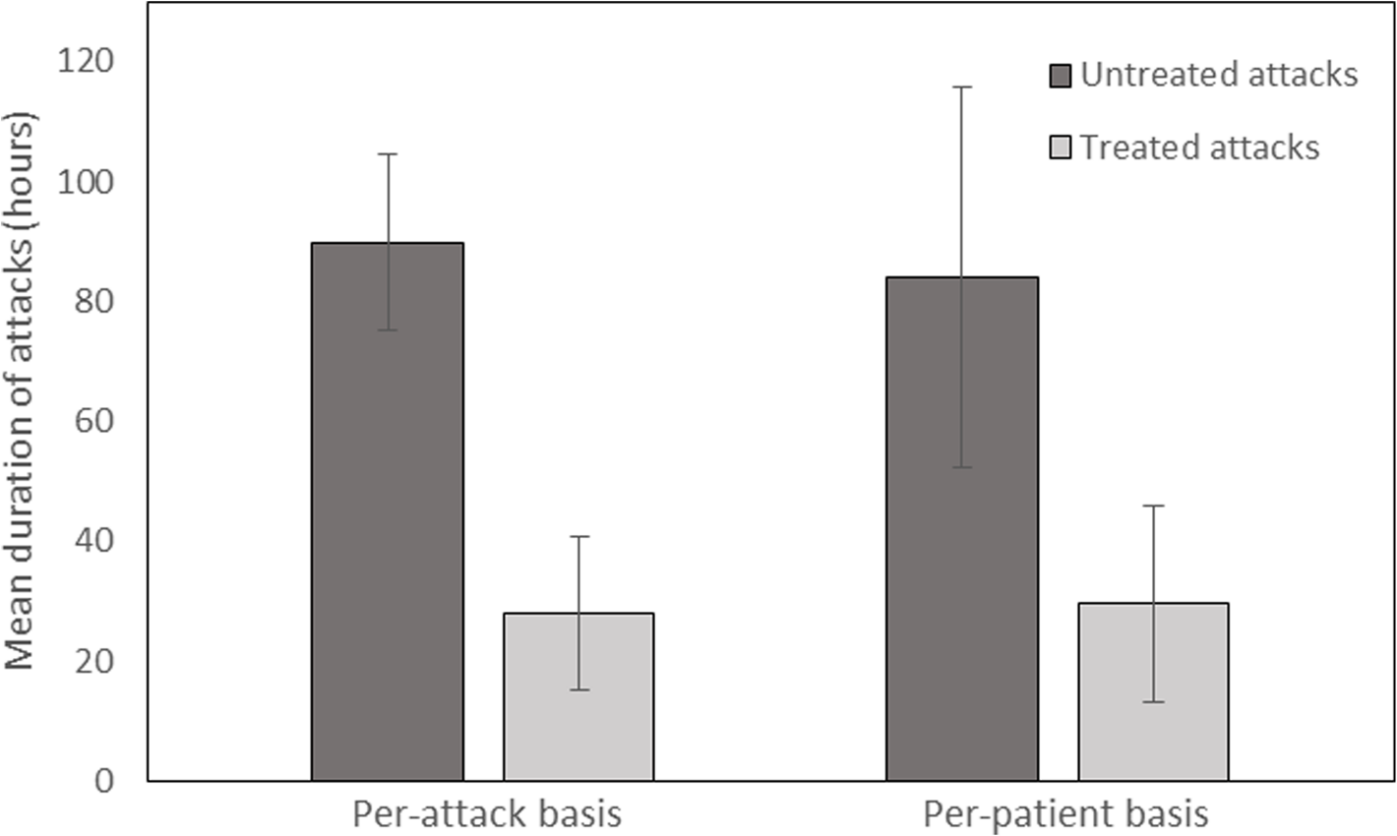
Efficacy of plasma-derived C1-inhibitor concentrate in acquired angioedema due to C1-inhibitor deficiency. Mean (SD) duration of untreated versus treated attacks is shown SD = standard deviation.

The mean (SD) duration of untreated attacks was 89.9 (± 14.8) hours over all attacks in the 32 patients and 84 (± 31.8) hours on a per-patient basis (mean of 32 averages). The mean (SD) duration of the treated attacks was 27.9 (± 12.9) hours over all attacks in the 32 patients and 29.6 (± 16.4) hours on a per-patient basis. (Fig. 2). Treatment with pdC1-INH concentrate shortened attacks by an average of 54.4 (± 32.8) hours (confidence interval (CI): 42.5, 66.2) (*P* < 0.0001) on a per-patient basis (i.e. by 64.8%).

All 32 patients responded to treatment, with 12 (37.5%) patients experiencing relief in 30 min or less. The average time to first relief after injection was 1.0 (± 3.3) hour in all 32 patients and 3.6 h (± 8.2) on a per-patient basis. Resolution of symptoms occurred on average 26.4 (± 12.6) hours after injection in all 32 patients and 26.7 (± 15.0) hours on a per-patient basis.

A total of 3553 (97.7%) of the 3636 attacks were effectively treated as assessed by the patient. Eighty-eight (2.3%) attacks in 8 patients did not or did not sufficiently respond to pdC1-INH treatment. Among the 8 non-responders were 4 patients with anti-C1-INH autoantibodies (described further below).

### Effect of time to injection on efficacy

The mean (SD) time between attack onset and injection was 1.5 (± 0.9) hours for the 32 patients and 3.0 (± 2.7) hours on a per-patient basis. Regression analysis showed that there was a linear association between the average time to complete resolution of symptoms and the average time to injection i.e. the earlier the attack was treated, the shorter the time between injection and resolution of symptoms (*P* = 0.0149). Sensitivity analysis, omitting 2 patients with extreme times to injection, confirmed the association (*P* = 0.0036). Similar regression analysis showed no clear association between the average shortening of attack duration and the time to injection (*P* = 0.0745).

### Effect of dose on efficacy

In order to analyze the effect of dose, a subgroup analysis of patients using either 500 U or ≥ 1000 U in at least 90% of their acute injections was performed (Table 3). Overall, the mean (SD) dose per-attack was 787 (± 442) U. The majority of patients received either 500 U (*n* = 13) or 1000 U (*n* = 9) in more than 90% of attacks; 1 patient received 1500 U and 1 patient received 2000 U. Patients in both dose subgroups had similar body weights and mean times between attack onset and injection (Table 3).

**Table 3:** Effect of dose on plasma-derived C1-inhibitor concentrate efficacy

	500 U ^a^ (*N* = 13)	≥ 1000 U ^a^ (*N* = 11)
	Mean (SD)	
Body weight, kg	68.8 (24.0)	70.8 (12.6)
Time between attack onset and injection, hours	2.3 (1.1)	3.3 (4.2)
Shortening of attack with treatment, hours	53.1 (25.9)	58.8 (40.1)
Time between injection and resolution of symptoms, hours	27.6 (16.3)	21.9 (11.3)

^a^dose received in at least 90% of administrations

SD = standard deviation

In the 500 U dose group, attack duration was shortened by a mean (SD) 53.1 (± 25.9) hours and in the ≥1000 U dose group by a mean (SD) 58.8 (± 40.1) hours. In the 500 U dose group, the time between injection and resolution of symptoms was 27.6 (± 16.3) hours and in the ≥1000 U dose group it was 21.9 (± 11.3) hours. However, no association between dose and shortening of attack duration (*P* > 0.1) or dose and time between injection and resolution of symptoms (P > 0.1) could be shown by linear regression.

### Effect of anti-C1-INH autoantibodies on efficacy

Eight AAE-C1-INH patients were positive for anti-C1-INH autoantibodies and 6 patients were negative. The remainder of patients were not tested for the presence of anti-C1-INH autoantibodies.

Attack duration was shortened by a mean (SD) 50.4 (± 38.2) hours in the 8 patients who tested positive for antibodies to C1-INH and by 58.9 h (± 36.0) in the 6 patients who tested negative. The mean shortening of attack duration for each individual patient with anti-C1-INH autoantibodies is shown in Table 4. The mean (SD) time between injection and resolution of symptoms was 31.5 (± 21.1) hours in the antibody positive patients and 29.3 (± 17.5) hours in the antibody negative patients.

**Table 4:** Treatment efficacy in acquired angioedema C1-inhibitor patients with anti-C1-INH autoantibodies (*N* = 8)

Patient	Gender (M/F)	Type of anti-C1-INH autoantibodies	Untreated attacks (n)	Mean duration of untreated attacks (h)	Treated attacks (n)	Mean duration of treated attacks ^a^ (h)	Mean shortening of attacks with treatment (h, (%))	Effectively treated attacks ^b^ (n (%))
1	F	IgG	124	72	820	9.5	62.5 (86.8)	820 (100)
2	M	IgG, IgM	208	60	13	79	−19 (−31.7)	1 (8.3)
3	M	IgG	648	84	12	41.5	42.5 (50.6)	12 (100)
4	M	IgG, IgM	79	96	16	26	70 (72.9)	16 (100)
5	F	IgA	89	84	24	38	46 (54.8)	22 (91.7)
6	M	IgG	59	96	303	24.25	71.75 (74.7)	303 (100)
7	M	IgG, IgM	144	120	71	11	109 (90.8)	70 (98.6)
8	F	IgG, IgM	283	72	70	51.5	20.5 (28.5)	68 (97.1)
Total	–	–	1634	–	1329	–	–	1246 (93.8)

^a^time between onset of attack and injection + time between injection and resolution of symptoms

^b^as assessed by the patient

*C1-INH* = C1-inhibitor; *F* = Female; *Ig* = Immunoglobulin; *M* = Male

Table 4 shows the efficacy in the subgroup of patients with anti-C1-INH autoantibodies. The 8 antibody-positive patients had a total of 2963 attacks: 1634 were untreated and 1329 were treated with pdC1-INH. In all 8 patients, pdC1-INH was effective in 1246 attacks (93.8%). In 4 of the 8 patients, pdC1-INH was not effective in 83 attacks. These 4 patients had 95 further attacks which were effectively treated with pdC1-INH.

Five of the 8 anti-C1-INH autoantibody positive patients (patients 1, 2, 3, 7, 8) had no associated disorders such as MGUS or malignant lymphoma. These patients experienced 986 attacks, 971 (98.5%) of which were effectively treated, as assessed by the patient.

Table 5 shows the treatment effect by dose in the subgroups of patients with and without anti-C1-INH autoantibodies. In 6 patients without anti-C1-INH autoantibodies, 344 (99.4%) of 346 attacks were treated effectively, with an average (SD) dose of 510.2 (± 69.1) U per effectively treated attack. In 8 patients with anti-C1-INH autoantibodies, 1246 (93.8%) of the 1329 attacks were treated effectively, with an average (SD) dose of 1238.4 (± 578.2) U per effectively treated attack. In 4 of the 8 anti-C1-INH autoantibody positive patients, 83 attacks failed to respond or did not sufficiently respond to pdC1-INH. Sixty-nine (83.1%) of those 83 attacks were treated with 500 U. A dose of 500 U did, however, effectively treat 33 (32.4%) of the total 102 attacks.

**Table 5:** Dose-dependent treatment effects in acquired angioedema C1-inhibitor patients without (*N* = 6) and with (*N* = 8) anti-C1-INH autoantibodies

	Patients without autoantibodies against C1-INH (*N* = 6)						Patients with autoantibodies against C1-INH (*N* = 8)					
pdC1-INH dose (U)	No. of patients	No. of attacks	Effectively treated patients	Effectively treated attacks	Non-effectively treated patients	Non-effectively treated attacks	No. of patients	No. of attacks	Effectively treated patients	Effectively treated attacks	Non-effectively treated patients	Non-effectively treated attacks
500	3	338	3	337	1	1	7	102	5	33	3	69
1000	4	8	3	7	1	1	6	907	5	900	3	7
1500	0	0	0	0	0	0	2	4	1	1	1	3
2000	0	0	0	0	0	0	4	315	3	311	1	4
3000	0	0	0	0	0	0	1	1	1	1	0	0
	-.	346	–	344	2	2	–	1329	–	1246	–	83

Note: The same patients may be represented as effectively treated and non-effectively treated

*C1-INH* = C1-inhibitor

## Discussion

The clinical features of our patients diagnosed with AAE-C1-INH were similar to those described in previous AAE-C1-INH studies [1, 4, 18]. In our institution, we found that the incidence of the condition was 1 patient with AAE-C1-INH for every 9.3 patients with HAE-C1-INH. In other studies, an incidence of 1:8.8 [1], 6% [19] and 10% [18] was reported. The clinical picture of our AAE-C1-INH patients differs from a large series of HAE-C1-INH patients previously described by us [20]. AAE-C1-INH patients have (1) a higher number of facial swellings (29.6% versus 1.6%), (2) a lower number of extremity swellings (25.7% versus 45.1%), and (3) a higher number of tongue swellings (1.8% versus 0.3%) than the HAE-C1-INH patients. The difference in the swelling patterns between AAE-C1-INH and HAE-C1-INH shows that a low level of functional C1-INH activity alone does not determine the swelling pattern. Furthermore, the number of patients with preceding erythema marginatum is lower in AAE-C1-INH patients (4.5%) than in HAE-C1-INH patients (30 to 60%) [21, 22].

In most patients in our series, onset of AAE-C1-INH occurred at age 40 years or older. However, there were a few patients who were less than 40 years when their AAE-C1-INH started. Among these were 3 patients with anti-C1-INH autoantibodies but no MGUS or malignant lymphoma. We conclude, therefore, that a diagnosis of AAE-C1-INH cannot be ruled out in patients less than 40 years old.

We found that AAE-C1-INH was associated with a variety of disorders including MGUS, malignant non-Hodgkin lymphoma, anti-C1INH autoantibodies, and other conditions. In some patients, there was no associated disorder identifiable. A total of 27.3% patients had an underlying lymphoma and in 75.0% of those patients the lymphoma was detected by monitoring the signs of AAE-C1-INH. This underscores the importance of early diagnosis of AAE-C1-INH. We also report that 25.0% of patients had a low-grade malignant lymphoma and approximately half of them had a splenic marginal cell lymphoma. This confirms that splenic marginal cell lymphoma is the most common type of underlying lymphoma in patients with AAE-C1-INH [23, 24].

MGUS and non-Hodgkin lymphoma are both B-cell lymphoproliferative disorders. MGUS may transform into plasmocytoma, Waldenström’s macroglobulinemia and other lymphoproliferative disorders. How lymphoproliferative disorders result in C1-INH deficiency is not exactly clear. Binding of C1-INH to the dysprotein of MGUS or directly to lymphoma tissue could lead to a low level of C1-INH. The role that anti-C1-INH autoantibodies play in C1-INH deficiency is less clear. A total of 11.4% of patients in our series had anti-C1-INH autoantibodies without MGUS, lymphoma or other associated disorder, even after a long observation period of 15.8 years. Our results show that MGUS may underlie AAE-C1-INH with and without anti-C1-INH autoantibodies and, also, that malignant lymphoma may underlie AAE-C1-INH in the presence or absence of dysproteins or anti-C1-INH autoantibodies. Some patients with AAE-C1-INH have only neutralizing autoantibodies to C1-INH and no other associated disorder. In the past, it was assumed that this was a separate type of AAE-C1-INH (AAE-C1-INH type 2) [25, 26]. The younger age at onset of angioedema described here could be a special feature of this subgroup of patients. However, the small number of patients needs to be considered. At present, it is not clear whether lymphoproliferative disorders (MGUS and lymphoma) and anti-C1-INH autoantibodies have one common pathogenic mechanism leading to C1-INH deficiency.

Our results show that pdC1-INH reduces the average attack duration by more than 60%. It is highly effective in resolving attacks, as evaluated by attack and by patient analysis, most notably if treatment is administered early in an attack. According to patients’ self-assessment, all patients responded well to pdC1-INH in nearly all of their attacks.

It has been reported that a few AAE-C1-INH patients have needed treatment with high doses of pdC1-INH or that some patients have become completely or partially resistant to this treatment [4]. Some of these patients had autoantibodies to C1-INH concentrate [1, 27]. In contrast, our results show that the vast majority (93.8%) of attacks in patients with anti-C1-INH autoantibodies responded well to pdC1-INH and that the response rate was similar to patients without anti-C1-INH autoantibodies (99.4% of attacks). Therefore, we conclude that patients with anti-C1-INH antibodies can respond to pdC1-INH. However, in some patients with anti-C1-INH autoantibodies, doses to effectively treat attacks need to be more than double that in patients without autoantibodies. In our series, 4 out of 8 patients with anti-C1-INH autoantibodies did not respond sufficiently to pdC1-INH treatment in some attacks, whereas in other attacks in the same patients, treatment with pdC1-INH was effective. A variation in the level of anti-C1-INH autoantibodies may be a reason for this. Repeated and long-term investigations of the anti-C1-INH autoantibody levels in AAE-C1-INH patients are needed to explain the relationship between autoantibody level and treatment dose.

Given the low prevalence of the disorder, this is a relatively large observational study examining the clinical characteristics of AAE-C1-INH. The study recruited a considerable number of patients and recorded the details of a large number of attacks treated with pdC1-INH over a long period of time. The study is limited by its observational and retrospective study design involving data retrieval from patient reports, which may have resulted in some bias. The fact that not all patients could be tested for anti-C1-INH autoantibodies also limits the results.

## Conclusions

In summary, our study demonstrated that the clinical symptoms of AAE-C1-INH are similar to those of HAE-C1-INH. Early diagnosis of AAE-C1-INH is important because of the risk of asphyxiation by laryngeal edema and because AAE-C1-INH is frequently associated with an underlying malignant disorder. AAE-C1-INH attacks can be treated with pdC1-INH, which is fast-acting and is highly effective in nearly all AAE-C1-INH attacks, including those in patients with anti-C1-INH autoantibodies.

## Methods

### Patients

Patients for this observational study were followed up at the AOSM from March 1986 to August 2017. Diagnosis of AAE-C1-INH was based on personal history of recurrent angioedema, no family history of angioedema and plasma examination of C1-INH, C4 and C1q. The absence of a C1-INH (*SERPING1)* genetic mutation confirmed diagnosis in some patients. The study was approved by the local ethics committee (Ethics Committee of the Landesärztekammer Rheinland-Pfalz, 837.413.13 (9098-F)) and all patients gave their informed consent to participate in the study.

### Study design

The clinical health records of 44 patients with AAE-C1-INH were reviewed retrospectively and patient characteristics noted. Thirty-two of these patients were treated for acute attacks with pdC1-INH. The efficacy variables were defined as follows: (1) the duration by which attacks were shortened after treatment, (2) the time between injection and resolution of symptoms, and (3) patient assessed treatment efficacy. The duration of treated attacks was compared with the duration of untreated attacks in the same individuals. Untreated attacks were defined as those which occurred before the first administration of pdC1-INH. Treated attacks were defined as those treated with pdC1-INH. Attack duration was defined as the time between attack onset and resolution of symptoms. Data on the time to injection and the type and duration of the attacks was recorded using standardized questionnaires. The course of the attacks was divided into first relief from symptoms and complete resolution of symptoms. In addition, patients evaluated the treatment effectiveness themselves during interviews and/or documented it in a patient diary. The following treatment evaluations were recorded by patients: treatment effective (responders) and treatment not or not sufficiently effective (non-responders).

### Treatment

The treatment group received intravenous, pasteurized pdC1-INH concentrate (Berinert® (CSL Behring, Marburg, Germany)) which was either self-injected or administered by the patient’s general practitioner, at their local hospital or at our department. The usual dose comprised 500 U of pdC1-INH, which corresponds to a C1-INH plasma activity of about 500 mL of fresh plasma. For treatment of skin swellings, 500 U pdC1-INH was recommended. If the patient had a body weight over 80 kg or if the patient felt that the 500 U dose was not sufficiently effective, 1000 U pdC1-INH was administered. Patients were counseled that mild skin swellings should not be treated. Mild skin swellings were defined as swellings limited to the back of one hand or foot or swellings at one extremity or at the trunk with a diameter of < 20 cm. Treatment was recommended for all facial and genital swellings. In skin swellings of the extremities, treatment was only recommended when the swellings were > 20 cm in diameter or if the whole extremity was affected or when the swelling of one extremity was followed by a swelling of another extremity or parts of the trunk within 24 h. Doses higher than 1000 U were administered only if the clinical response was not sufficient. All patients received vaccination for hepatitis B virus.

### Laboratory methods

C1-INH function was determined using the chromogenic substrate C2H5CO-Lys(e-Cbo)-Gly-Arg-pNA (Immunochrom C1-INH, Technoclone, Vienna, Austria). Normal functional levels of C1-INH were defined as 70–130%. Antigenic levels of C1-INH, C4 and C1q were quantified by radial immunodiffusion. Plasma protein levels were considered normal if they fell within the following ranges: C1-INH = 15.4–33.8 mg/dL, C4 = 16.4–31.3 mg/dL, and C1q = 0.1–0.25 g/L. Autoantibodies to C1-INH were measured as previously described [26].

### Statistical analysis

#### Patient characteristics

The following parameters were analyzed with univariate statistics (mean and SD) or frequency tables, as appropriate, for all patients and patients treated with pdC1-INH: baseline and demographic characteristics, associated disorders, monoclonal gammopathy antibodies, anti-C1-INH autoantibodies, prior and concomitant treatment, number of stays at hospital due to angioedema, number of stays in the intensive care unit, number of intubations, number of untreated swellings, and the average duration of untreated attacks. Univariate statistics for C1-INH function, C1-INH protein, C4 and C1q at first visit in AOSM were also generated for all patients and for patients treated with pdC1-INH.

#### Plasma-derived C1 inhibitor concentrate treatment

Univariate statistics for single doses of pdC1-INH were applied for both per-attack analysis and per-patient (on average doses) analysis. The number of attacks treated with pdC1-INH, the duration of pdC1-INH treatment, and the age at last pdC1-INH injection were analyzed with univariate statistics in a per-patient analysis. The number of attacks at different sites and the number of attacks treated with different doses of pdC1-INH were analyzed with a frequency table and frequency graph statistics in a per-attack analysis. In addition, a per-patient analysis was generated to show the number patients who preferably (i.e. in 90% or more of their attacks) received a special single dose of pdC1-INH.

For the time between onset of attack and injection, the time between injection and first sign of efficacy, the time between injection and complete resolution of symptoms, and the average shortening of attacks with treatment, univariate statistics were generated both in a per-patient and in a per-attack analysis.

#### Efficacy variables

The original dataset included data on the average duration of attacks with and without treatment with pdC1-INH for each patient as well as the number of attacks for each patient. The primary analysis on duration of attack was, therefore, a per-patient analysis which provided statistics on the average attack durations over all treated patients. In addition, a descriptive analysis on a per-attack basis was done by weighting the individual average duration of attacks by the individual number of attacks (untreated and treated).

Univariate statistics were applied for the average duration of attack treated with pdC1-INH and for the average duration of untreated attacks in both a per-patient and in a per-attack analysis. For the average shortening of the duration of attacks the following statistics were generated: univariate statistics, a two-sided Wilcoxon signed rank test (hypothesis: no shortening), and a 90% CI in a per-patient analysis.

In order to investigate the association between the time between injection and resolution of symptoms and the time between onset of attack and injection, linear regression analysis with graphical representation was generated. A similar regression analysis was performed for the association between average shortening of attacks and the time to injection, the association between shortening of attacks and the preferred single dose and the association between time to resolution of symptoms and the preferred single dose.

The percentage of effectively treated attacks, as assessed by the patient, was calculated for the total number of treated attacks, anti-C1-INH antibody positive patients, anti-C1-INH antibody positive patients with MGUS, and anti-C1-INH antibody negative patients. All analyses were performed using Excel or SAS.

## Abbreviations

AAE-C1-INH: acquired angioedema due to C1-inhibitor deficiency
AOSM: angioedema outpatient clinic at the Department of Dermatology, University of Mainz, Germany
C1-INH: C1 inhibitor
CI: confidence interval
HAE: hereditary angioedema
MGUS: monoclonal gammopathy of undetermined significance
pdC1-INH: plasma-derived C1-INH concentrate
